# Impact of a tailored, department-specific antimicrobial stewardship team intervention based on AWaRe guidelines: a single-center, cohort, interrupted-time series study

**DOI:** 10.1186/s40780-025-00525-3

**Published:** 2025-12-25

**Authors:** Yusuke Yagi, Yu Arakawa, Yukihiro Hamada, Yuka Yamagishi

**Affiliations:** 1 Department of Infection Prevention and Control, Kochi Medical School Hospital, 185-1, Kohasu, Oko-cho,, Nankoku city,, Kochi 783-8505 Japan; 2 Department of Pharmacy, Kochi Medical School Hospital, 185-1, Kohasu, Oko-cho, Nankoku city, Kochi 783-8505 Japan; 3 Department of Clinical Infectious Diseases, Kochi Medical School, Kochi University, 185-1, Kohasu, Oko-cho, Nankoku city, Kochi 783-8505 Japan

**Keywords:** Antimicrobial resistance, Antimicrobial stewardship, Oral outpatient antibiotic, Defined daily doses, Days of therapy, *Escherichia coli* susceptibility

## Abstract

**Background:**

The global crisis of antimicrobial resistance (AMR) necessitates robust antimicrobial stewardship. The World Health Organization’s (WHO) Access, Watch, Reserve (AWaRe) classification is a key tool for guiding antibiotic prescribing to combat AMR. The WHO’s global target was to have at least 60% of antibiotic consumption fall into the Access category by 2023. This study evaluated the effectiveness of localized interventions toward this goal.

**Methods:**

This retrospective, longitudinal study analyzed oral outpatient antibiotic prescriptions at Kochi Medical School Hospital from April 2022 to June 2025. Data included defined daily doses (DDDs), days of therapy (DOTs), and AWaRe classifications. Based on a preceding analysis, the antimicrobial stewardship team (AST) implemented targeted, department-specific educational interventions starting in April 2024. The impact was evaluated using seasonally adjusted time-series analysis and regression models. The study also included a survey of *Escherichia coli* susceptibility to levofloxacin, ciprofloxacin, and sulfamethoxazole/trimethoprim from outpatient urinary isolates.

**Results:**

The AST intervention led to a statistically significant increase in the usage proportion and DDDs of Access antibiotics (+ 2.92%, *p* = 0.005 and + 376.5 DDDs, *p* = 0.001, respectively), with a corresponding decrease in Watch antibiotics (-3.00%, *p* = 0.003). However, DOTs did not significantly change, indicating the intervention primarily impacted drug choice, not treatment duration. Top prescribed agents were clarithromycin (24.2%), sulfamethoxazole/trimethoprim (20.7%), and rifaximin (10.7%). *E. coli* susceptibility to levofloxacin and ciprofloxacin was low (< 80% overall), particularly in extended-spectrum β-lactamase (ESBL)-producing strains (~ 10%). Sulfamethoxazole/trimethoprim susceptibility remained high overall (≥ 80%) but was low (< 80%) for ESBL-producing strains.

**Conclusions:**

AST interventions successfully shifted outpatient prescribing from Watch to Access antibiotics, demonstrating that targeted interventions can modify prescribing behavior. However, challenges remain, such as addressing treatment duration and achieving broader goals. The observed resistance patterns highlight the critical need for prescribers to consult local antibiograms. Future strategies require a more comprehensive approach, including real-time alerts and improved diagnostics, to further optimize antibiotic use and combat AMR.

**Supplementary Information:**

The online version contains supplementary material available at 10.1186/s40780-025-00525-3.

## Background

The widespread and often injudicious use of antibiotics has fueled a global crisis of antimicrobial resistance (AMR), threatening the efficacy of these life-saving medicines [1]. In response, global health authorities have increasingly emphasized the implementation of effective antimicrobial stewardship programs [1]. A cornerstone of these efforts is the World Health Organization’s (WHO) Access, Watch, Reserve (AWaRe) classification, a framework designed to guide antibiotic prescribing and combat AMR at a systemic level [2, 3]. This classification categorizes antibiotics into three groups based on their resistance potential and importance to medicine [3]. The Access group includes narrow-spectrum, first-choice agents; the Watch group contains broader-spectrum, higher-risk antibiotics; and the Reserve group consists of last-resort agents for multi-drug resistant infections [4].

The AWaRe framework is a critical tool for monitoring antibiotic consumption and evaluating stewardship policies. In 2017, the WHO established a global target for antibiotic use, urging countries to ensure that at least 60% of their total antibiotic consumption falls within the Access category by 2023 [5]. In alignment with this strategy, the Japanese government has adopted this 60% target and since promoted initiatives to encourage institutions to achieve a high proportion of Access antibiotic use [6].

However, translating these national goals into effective, localized action remains a challenge. The global prevalence of extended-spectrum β-lactamase (ESBL)-producing *Escherichia coli* is increasing in both healthcare and community settings, posing a major issue for treating common infections [7]. While educational interventions on AWaRe-based prescribing have been shown to be effective [8, 9], the impact of interventions that are meticulously tailored to the unique prescribing patterns of specific outpatient departments remains poorly understood. This study aimed to evaluate a department-specific antimicrobial stewardship intervention within a Japanese university hospital. This granular approach serves to provide detailed insights into local prescribing behaviors and the specific challenges of implementing stewardship programs, complementing broader, less-targeted intervention studies.

## Methods

### Study design

This study adopted a retrospective, longitudinal study design to investigate the prescribing trends of oral antibiotics for outpatients. The investigation period for prescribing trends was from April 2022 to June 2025. To assess the impact of antimicrobial stewardship interventions, we conducted a time-series analysis of the prescribing trends from January 2023 to June 2025. AST interventions began in April 2024, and their content and methods were developed based on the analysis of prescribing patterns observed between April 2022 and March 2024. The effectiveness of these interventions was evaluated by analyzing changes in prescribing trends over time.

### Data collection

Data was collected from a comprehensive claims database, which included information on all outpatient visits in Kochi Medical School Hospital. The database contained detailed information on diagnoses, coded according to the International Classification of Diseases, 10th Revision (ICD-10), as well as patient demographics and prescription details. The drugs were classified into three categories using the Anatomical Therapeutic Chemical (ATC) classification system [10] and the AWaRe classification proposed by Sharland M et al. [11].

### Variable definitions

Antimicrobial consumption was measured using both defined daily doses (DDDs) and days of therapy (DOTs). DDDs were calculated according to the standard definitions provided by the WHO Collaborating Centre for Drug Statistics Methodology [12]. DOTs were defined as the number of days that a patient received at least one dose of an antibiotic, irrespective of dosage, as described by the Centers for Disease Control and Prevention (CDC) [13]. DDDs and DOTs were calculated using Eq. 1 and Eq. 2, respectively.

1 $$\:DDDs\:=\:TDD\left(g\right)\:/\:DDD\:\left(g\right)$$

TDD: total daily dose of antibiotics

DDD: defined daily dose of antibiotics

2 $$\:DOTs\:=\:{\sum\:}_{i=1}^{n}{d}_{i}\:\left(days\right)\:$$

n: total number of antibiotic types

d_i_: number of days of administration of the i-th antibiotic

DOTs: days of therapy

The usage proportion was defined as the percentage of each antibiotic’s DDDs relative to the total DDDs. The cumulative usage proportion was defined as the running total of these usage proportions, sorted in descending order.

### Primary outcome

The primary outcome of this study was the change in the usage proportion of Access and Watch antibiotics, following the implementation of the AST interventions.

### Antimicrobial susceptibility

We examined antimicrobial resistance in outpatient urinary tract infections (UTIs) caused by *Escherichia coli* and ESBL-producing *E. coli*. Our focus on levofloxacin, ciprofloxacin, and trimethoprim-sulfamethoxazole was motivated by the high global prevalence of UTIs [14] and the WHO’s prioritization of these pathogens and antibiotics for surveillance [15]. *Escherichia coli* susceptibility to levofloxacin, ciprofloxacin, and trimethoprim-sulfamethoxazole from outpatient urinary isolates was assessed. To avoid duplicates, only the first isolates from patients that were not receiving antibiotic therapy at the time of specimen collection during the study period were included, in accordance with Clinical and Laboratory Standards Institute (CLSI) recommendations [16]. Extended-spectrum β-lactamase (ESBL) production status (ESBL-producing vs. non-ESBL-producing) was determined following CLSI phenotypic confirmation guidelines [16]. Standardized antimicrobial susceptibility testing was performed using the microdilution method and automated systems validated per CLSI M100 standards (31st ed., 2021) [17, 18]. Susceptibility results (susceptible, intermediate or resistant) were interpreted using the most recent CLSI breakpoints (31st ed., 2021) [17, 18]. Susceptibility rates were calculated separately for levofloxacin, ciprofloxacin, and sulfamethoxazole/trimethoprim in ESBL-producing vs. non-ESBL-producing groups.

### Statistical analysis

This study used regression analyses to assess an intervention’s impact on usage proportion, DDDs, DOTs, and the number of outpatients who were prescribed antibiotics. Ordinary least squares regression was used to establish baseline models, followed by Durbin–Watson tests for first-order autocorrelation [19]. If values fell outside 1.5–2.5, indicating autocorrelation, Prais–Winsten estimation was applied using an estimated ⍴-value [20]. To account for seasonality, which is a common factor in antibiotic consumption, an intervention dummy variable and 11 dummy variables representing the months of the year were included in all models. The coefficient of determination (R²) was calculated to measure explained variance. The time series analysis was performed using R statistical software version 4.4.3 (https://www.r-project.org/). Antimicrobial susceptibility data from the three fiscal years, based on monthly aggregate rates, were compared using the Kruskal–Wallis test. A p-value of < 0.050 was considered statistically significant.

## Results

### Outpatient oral antibiotic prescription trends

Prescription trends for outpatient oral antibiotics were analyzed to identify the most commonly used agents (Table 1). The data revealed that the top four antibiotics accounted for a significant portion of prescriptions, with a cumulative contribution of approximately 63.4%. Clarithromycin (24.2%) and rifaximin (10.7%), both classified as Watch antibiotics, were among the most frequently prescribed. The Access antibiotic, sulfamethoxazole/trimethoprim (20.7%) and the Watch antibiotic, levofloxacin (7.8%) also demonstrated high usage rates. Additionally, the analysis showed that roxithromycin (7.0%) and minocycline (5.3%), both classified as Watch antibiotics, were the sixth and seventh most used, respectively. In terms of therapeutic classification, macrolides were highly prominent, and the largest usage of rifaximin was found in gastroenterology departments, accounting for nearly all its use. This concentration of antibiotic use in a small number of drugs highlighted key patterns in outpatient prescribing practices.

**Table 1:** Actual use of oral antibiotics in outpatients (April 2022 to March 2024)

Generic name	AWaRe classification	ATC code	DDD	Usage proportion	cumulative usage proportion	DDDs	DOTs	Actual results by department (%): Over 5%
Clarithromycin	Watch	J01FA09	0.5	24.2	24.2	56,474	67,337	Respiratory and Allergy (66.4%), Otolaryngology and Head and Neck Surgery (8.3%), and Nephrology and Endocrinology (5.5%)
Sulfamethoxazole/trimethoprim	Access	J01EE01	4	20.7	44.9	48,312	192,841	Nephrology and Endocrinology (41.4%), Respiratory and Allergy (14.6%), and Hematology (13.1%)
Rifaximin	Watch	A07AA11	0.6	10.7	55.6	25,024	14,464	Gastroenterology (99.9%)
Levofloxacin	Watch	J01MA12	0.5	7.8	63.4	18,343	19,020	Urology (18.0%), Orthopedics (15.9%), Breast tumor surgery (12.5%), and Oncology (8.7%)
Doxycycline	Access	J01AA02	0.1	7.4	70.8	17,297	14,362	Dermatology (88.2%) and Respiratory and Allergy (5.5%)
Roxithromycin	Watch	J01FA06	0.3	7.0	77.8	16,302	16,857	Dermatology (90.7%) and Respiratory and Allergy (8.7%)
Minocycline	Watch	J01AA08	0.2	5.3	83.1	12,298	15,190	Dermatology (46.3%), Orthopedics (16.8%), and Oncology (10.5%)
Erythromycin	Watch	J01FA01	1	5.1	88.2	11,902	33,656	Respiratory and Allergy (74.7%)
Amoxicillin	Access	J01CA04	1.5	2.3	90.5	5,394	10,849	Otolaryngology and Head and Neck Surgery (16.2%), Respiratory and Allergy (14.4%) and Cardiology (12.7%)
Cefaclor	Watch	J01DC04	1	2.2	92.6	5,063	11,584	Urology (39.5%) and Dermatology (21.3%)
Amoxicillin/clavulanic acid	Access	J01CR02	2.25	1.0	93.6	2,312	4,386	Respiratory and Allergy (32.8%) and Dermatology (10.0%)
Sitafloxacin	Watch	J01MA21	0.1	0.9	94.6	2,200	2,207	Respiratory and Allergy (76.1%) and Urology (11.1%)
Cefcapene	Watch	J01DD17	0.45	0.8	95.3	1,801	2,728	Urology (65.7%), Gastroenterology (7.7%), and Dermatology (26.2%)
Cefalexin	Access	J01DB01	2	0.8	96.1	1,794	4,316	Dermatology (20.1%), Cardiology (19.6%), and Obstetrics and Gynecology (16.6%)
Azithromycin	Watch	J01FA10	0.3	0.7	96.8	1,673	1,076	Respiratory and Allergy (41.5%), Nephrology and Endocrinology (14.8%), Pediatrics (12.1%), and Infectious Diseases (10.7%)
Sultamicillin	Access	J01CR04	1.5	0.6	97.4	1,396	2,331	Dermatology (24.5%), Otolaryngology and Head and Neck Surgery (21.1%), and Orthopedics (20.8%)
Clindamycin	Access	J01FF01	1.2	0.3	97.8	792	1,794	Orthopedics (80.5%), Nephrology and Endocrinology (10.8%)
Cefpodoxime	Watch	J01DD13	0.4	0.3	98.1	699	1,635	Urology (41.8%), Dermatology (28.5%), and Pediatrics (18.7%)
Fosfomycin	Watch	J01XX01	3	0.3	98.4	692	1,289	Cardiology (46.3%), Urology (19.1%)
Lascufloxacin	Watch	J01MA25	0.075	0.3	98.6	660	659	Respiratory and Allergy (52.6%) and Otolaryngology and Head and Neck Surgery (47.4%)
Cefdinir	Watch	J01DD15	0.6	0.3	98.9	608	1,262	Neurology (39.5%), Dermatology (21.3%), and Urology (12.7%)
Kanamycin	Watch	A07AA08	3	0.2	99.1	572	1,144	Gastroenterology (100%)
Garenoxacin	Watch	J01MA19	0.4	0.2	99.4	528	538	Respiratory and Allergy (41.2%) and Otolaryngology and Head and Neck Surgery (34.5%)
Cefditoren	Watch	J01DD16	0.4	0.2	99.6	503	693	Obstetrics and Gynecology (16.6%), Otolaryngology and Head and Neck Surgery (34.5%), and Pediatrics (18.7%)
Metronidazole	Access	P01AB01	2	0.2	99.7	362	811	Obstetrics and Gynecology (60.5%), Surgery (16.3%), and Infectious Diseases (13.3%)
Faropenem	Reserve	J01DI03	0.75	0.1	99.9	298	389	Urology (64.2%) and Dermatology (32.0%)
Ciprofloxacin	Watch	J01MA02	1	0.1	99.9	129	237	Neurology (31.7%), Obstetrics and Gynecology (16.1%), and Urology (12.6%)
Prulifloxacin	Watch	J01MA17	0.6	0.04	99.97	97	89	Gastroenterology (82.6%) and Dermatology (9.9%)
Linezolid	Reserve	J01XX08	1.2	0.03	100	81	81	Orthopedics (43.8%), Urology (33.3%), and Plastic Surgery (16.2%)
Tosufloxacin	Watch	J01MA22	0.45	0.03	100	60	100	Pediatrics (83.7%) and Otolaryngology and Head and Neck Surgery (16.7%)
Fidaxomicin	Watch	A07AA12	0.4	0.004	100	10	10	Gastroenterology (100%)
Tebipenem	Watch	J01DH06	0.56	0.0003	100	1	3	Pediatrics (100%)
Tedizolid	Reserve	J01XX11	0.2	0	100	0	0	No usage record

AWaRe, Access, Watch Reserve; ATC, Anatomical Therapeutic Chemical; DDDs, defined daily doses; DOTs, days of therapy

### AST intervention contents

The interventions focused on antibiotics that constituted over 90% of the cumulative usage proportion (Table 1), as identified in a preceding analysis. The AST, which comprised an infectious disease physician and a clinical pharmacist, developed department-specific interventions. The interventions were delivered through multiple formats. Initially, face-to-face group presentations were held for each target department to explain the analysis results and propose changes. This was followed by quarterly feedback reports emailed to each department, showing their prescribing trends compared to the hospital averages. The AST was also available for individual consultations. Adherence to the recommendations was monitored via monthly reviews of prescription data. AST interventions focused on improving prescribing practices across multiple departments. For respiratory and allergy patients, the team recommended reducing the duration of prescriptions and avoiding long-term administration of clarithromycin. Dermatology and oncology departments received suggestions to change from less-supported antibiotics to guideline-recommended agents, such as switching to doxycycline. In oncology, an intervention shortened the clinical path for levofloxacin from seven to four days for some patients. Otorhinolaryngology and pediatrics interventions advised selecting antibiotics based on disease severity and ceasing prolonged administration for chronic conditions. Prophylactic use was also a target, with the AST advising gastroenterology not to administer rifaximin for minimal hepatic encephalopathy. Overall, the interventions were tailored to specific departments and aimed at optimizing duration, drug choice, and dosage to improve antimicrobial stewardship (Table 2).

**Table 2:** Practice and effects of antimicrobial stewardship team awareness activities

Department	Antibiotics	Purpose of use	Proposal based on insights from antimicrobial stewardship team	Department specific measures
Respiratory and Allergy	Erythromycin	Diffuse panbronchiolitis	Avoid long-term administration, although the need is high [29].	Reduce the number of prescribing days while conducting periodic evaluation of efficacy.
	Clarithromycin			Strengthen cooperation between medical institutions and prescribe at collaborating medical institutions.
Dermatology	Roxithromycin	Inflammatory rash	Change to doxycycline, which is the most highly recommended on the guideline [36].	Prescribe doxycycline to a new patient.
	Minocycline			
Otorhinolaryngology Head and Neck Surgery Pediatrics	Erythromycin Clarithromycin	Sinusitis Otitis media	Depending on the severity of the disease, do not administer for mild disease, amoxicillin for moderate disease, and amoxicillin or amoxicillin/clavulanic acid for severe disease [28]. In addition, prolonged administration for exudative otitis media or chronic sinusitis should be stopped [28].	Using the lower end of the guideline’s recommended dose range for erythromycin or clarithromycin in less severe cases. Avoid long-term administration.
Gastroenterology	Rifaximin	Hepatic encephalopathy	No prophylactic administration for minimal hepatic encephalopathy and subclinical hepatic encephalopathy [33].	Consider prescribing at collaborating medical institutions through the coordination of medical diagnoses.
Oncology	Levofloxacin	Febrile neutropenia	For patients at low risk of severe disease, switch to amoxicillin/clavulanic acid + ciprofloxacin, which is highly recommended [35].	Change the number of days in the clinical path from seven to four days.
	Minocycline	Acneiform rash	Change to doxycycline, which has a stronger level of evidence in the guidelines [37].	Change the minocycline on the clinical path to doxycycline.
Breast tumor surgery	Levofloxacin	Febrile neutropenia	Consider administration according to the duration of neutropenia [35].	Perform a risk assessment for neutropenia and prescribe only to patients who need it.
			Change to amoxicillin/clavulanic acid + ciprofloxacin, which is highly recommended for therapeutic purposes [35].	

### Verification of AST intervention impact

**Fig. 1:**
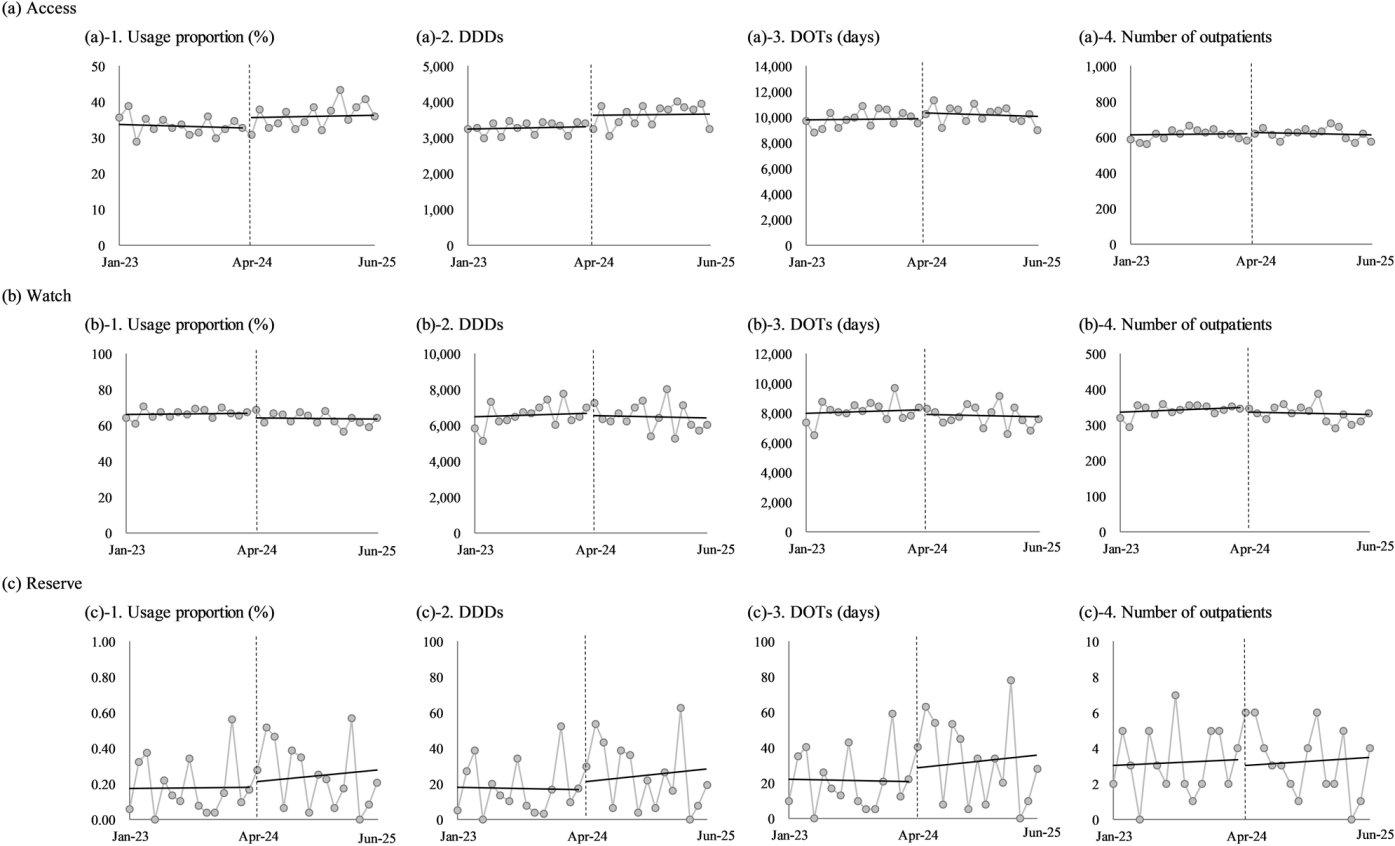
Trends in monthly consumption of oral antibiotics for outpatients from January 2023 to June 2025. The analysis was stratified by the WHO AWaRe classification: (**a**) Access, (**b**) Watch, and (**c**) Reserve antibiotics. The metrics shown for each category are: (1) usage proportion (%), (2) Defined Daily Doses (DDDs), (3) Days of Therapy (DOTs), and (4) number of outpatients. The vertical dashed line indicates the start of the antimicrobial stewardship team (AST) intervention in April 2024. Solid lines represent the regression lines of the time-series analysis

The results of the pre- and post-intervention analysis are shown in Table 3; Fig. 1. For Access antibiotics, the intervention had a statistically significant positive effect on usage proportion and DDDs, increasing them by 2.92% (*p* = 0.005) and 376.5DDDs (*p* = 0.001), respectively. The high R² values (R²=0.69 and R²=0.59, respectively) indicated that the models explained a substantial portion of the variance. No significant effects were observed for DOTs (*p* = 0.115) or the number of outpatients prescribed antibiotics (*p* = 0.701). For Watch antibiotics, the intervention significantly decreased usage proportion by 3.00% (*p* = 0.003) and, the model’s fit was good, as it explained 70% of the data’s variability (R²=0.70). These models did not detect any statistically significant changes post-intervention. For Reserve antibiotics, no statistically significant changes were found for any variable (all p-values > 0.05). The R² values were modest (R² ranged from 0.38 to 0.50), suggesting that the models could only explain a smaller portion of the variance in this category.

**Table 3:** Results of pre/post intervention analysis and regression models

Classification	Variable	Mean (Pre)	SD (Pre)	Mean (Post)	SD (Post)	Regression model	Intervention coefficient	*p*-value	*R* ^2^	DW stat.	⍴-value
(a) Access	(a)-1 Usage proportion	33.3	2.59	36.1	3.45	OLS	2.92	0.005	0.69	2.14	-
	(a)-2 DDDs	3276.8	165.2	3629.3	304.5	OLS	376.5	0.001	0.59	2.29	-
	(a)-3 DOTs	9853.7	624.3	10198.7	663.3	OLS	362.6	0.115	0.55	2.36	-
	(a)-4 Number of outpatients	611.9	29.2	619.7	31.9	Prais–Winsten	4.39	0.701	0.46	1.20	0.380
(b) Watch	(b)-1 Usage proportion	66.5	2.57	63.7	3.41	OLS	-3.00	0.003	0.70	2.20	-
	(b)-2 DDDs	6577.2	668.3	6471.7	769.4	OLS	-74.1	0.749	0.58	2.29	-
	(b)-3 DOTs	8125.1	724	7799.3	707.7	OLS	-332.0	0.160	0.60	2.26	-
	(b)-4 Number of outpatients	341.3	17.2	332.1	24.9	OLS	-10.8	0.186	0.46	1.91	-
(c) Reserve	(c)-1 Usage proportion	0.18	0.16	0.24	0.18	OLS	0.080	0.228	0.40	1.87	-
	(c)-2 DDDs	17.5	14.9	24.8	18.9	OLS	9.48	0.140	0.48	1.75	-
	(c)-3 DOTs	21.2	16.6	32.0	23.6	OLS	13.3	0.086	0.50	1.88	-
	(c)-4 Number of outpatients	3.2	1.9	3.27	1.94	OLS	0.100	0.898	0.38	1.88	-

DDDs, defined daily doses; DOTs, days of therapy; SD, standard deviation; OLS, Ordinary Least Squares; DW stat., Durbin–Watson_statistic

### Antimicrobial susceptibility

**Fig. 2:**
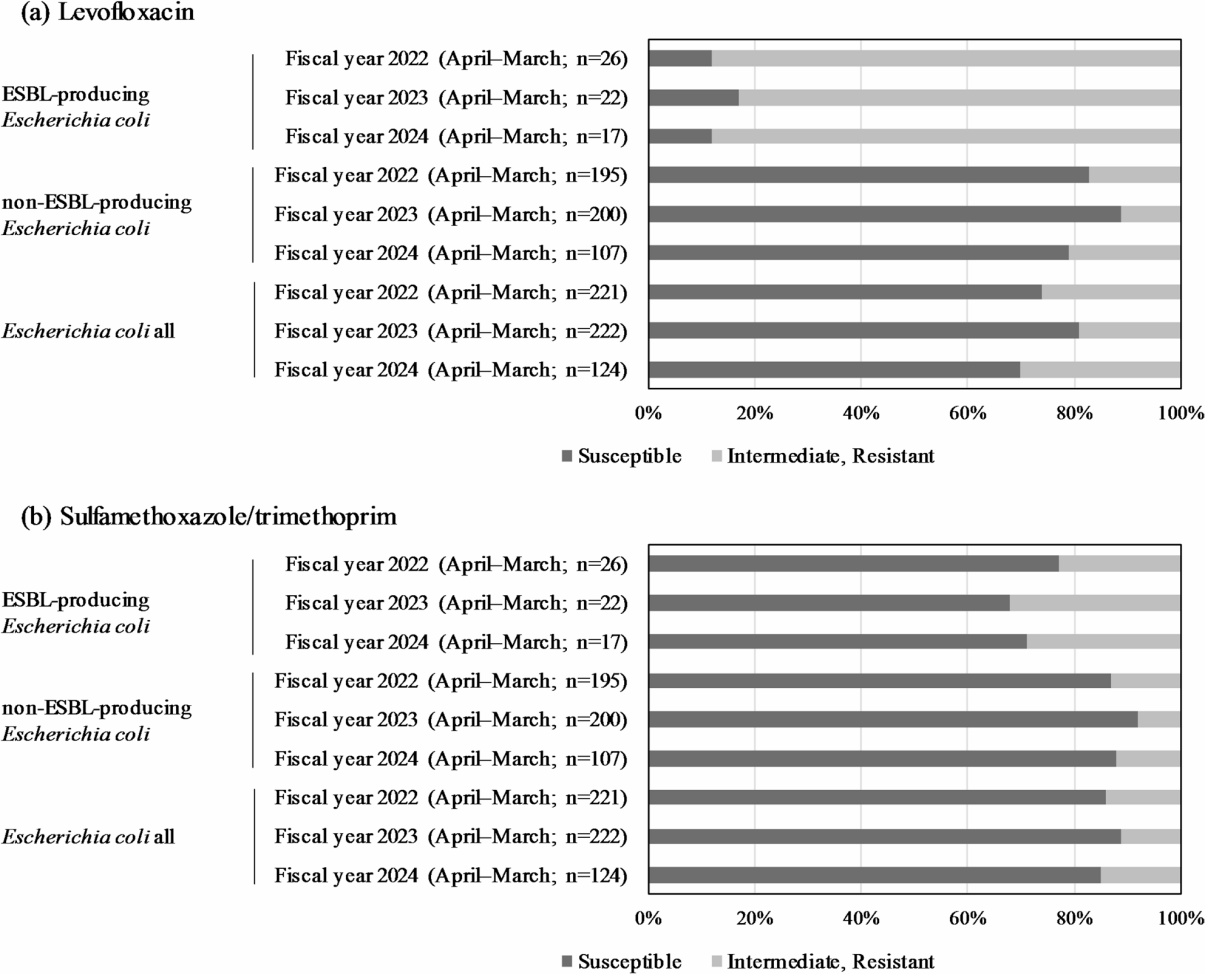
Antimicrobial susceptibility results for *Escherichia coli* (**a**) drug susceptibility rate of levofloxacin against *E. coli*, (**b**) drug susceptibility rate of sulfamethoxazole/trimethoprim against *E. coli.* A Kruskal–Wallis test was performed on the three groups over a three-year period. No significant differences in susceptibility to (**a**) and (**b**) were found

Levofloxacin susceptibility remained high in non-ESBL *E. coli* (81–89%) but low in ESBL-producing strains (11–17%). Similar results were observed for ciprofloxacin (data not shown). Sulfamethoxazole/trimethoprim susceptibility showed a similar pattern, with non-ESBL strains having significantly higher susceptibility (85–89%) than their ESBL-producing counterparts (8–13%) over the three-year period. No statistically significant difference in susceptibility to either drug was observed over the three-year period (Fig. 2).

## Discussion

This single-center, retrospective study investigated the impact of a department-specific AST intervention on outpatient antibiotic prescribing. We assessed the shift from Watch to Access antibiotics and analyzed high-volume drug usage and resistance patterns. The findings offer a novel, localized perspective on outpatient antibiotic use and resistance in a Japanese healthcare setting.

Our AST interventions were effective in influencing prescribing behaviors, as evidenced by a statistically significant increase of 2.92% in the proportion of Access antibiotics (*p* = 0.005) and a corresponding decrease of 3.00% in Watch antibiotics (*p* = 0.003). This outcome, further supported by a substantial increase of 376.5DDDs in Access antibiotic (*p* = 0.001), demonstrates that a tailored, educational approach, focusing on specific departments and their most frequently used agents, can effectively modify prescribing habits. While our results did not reach the national target of 60% for the Access antibiotic proportion, the significant improvement represents a positive step. This magnitude of change, achieved over a relatively short period, demonstrates that targeted local interventions can contribute substantially toward meeting national goals and may help curb the emergence of resistance by promoting first-choice agents. Furthermore, the high R² values observed in our fixed-effects models (R² >0.50 for all primary outcomes) demonstrated that after controlling for seasonality, the intervention strongly accounted for improving prescription trends. The remaining influences, which we did not control for, still impacted trends; these confounding factors may include changes in local disease epidemiology [21], evolving national/international treatment guidelines [22], and antibiotic supply chain disruptions [23] (although we confirmed the absence of critical shortages for Access and Watch antibiotics during the study period). The non-significant changes in DOTs and the number of outpatients prescribed antibiotics suggested that our interventions primarily impacted the type of antibiotic prescribed rather than the overall volume or duration of treatment, highlighting a key area for future improvement. This may be because changing the drug of choice is often perceived as a more straightforward action for prescribers than altering empirically established treatment durations, especially for chronic conditions. Overcoming this inertia may require more intensive interventions, such as real-time alerts in the electronic tools or mandatory reviews for long-term prescriptions [9]. Conversely, our analysis found no statistically significant changes in the usage of Reserve antibiotics across any of the measured indicators. This lack of observable change is likely due to their limited indications in an outpatient setting [24, 25] or the high data variability and resulting estimation instability from the limited sample size in this group.

The analysis of specific high-volume antibiotics provided critical insights. Clarithromycin was the most prescribed drug, accounting for 24.2% of all prescriptions, with its prevalence likely driven by empirical treatment for respiratory infections [26] and its non-antimicrobial, anti-inflammatory properties in chronic airway diseases like bronchiectasis [27]. The extended durations often recommended for these non-infectious conditions can lead to prolonged treatment, which necessitates a regular, specialist-led re-evaluation of its necessity and duration to mitigate resistance development [28, 29]. The second most used agent, sulfamethoxazole/trimethoprim, accounted for 20.7% of prescriptions and is primarily used for *Pneumocystis jirovecii* pneumonia prophylaxis in immunocompromised patients [30]. Our finding that its susceptibility rate for *E. coli* was maintained at ≥ 80% is reassuring, but this rate dropped to below 80% for ESBL-producing strains. This is a finding of particular clinical importance, as the increasing prevalence of community-acquired ESBL-producing *E. coli* in Japan necessitates vigilance [31]. This resistance pattern underscores the need for prescribers to consult local antibiograms and consider alternative drugs when treating infections, especially urinary tract infections, where *E. coli* is a common causative agent [32]. The third most common antibiotic, rifaximin, accounted for 10.7% of prescriptions and was almost exclusively used in gastroenterology for hepatic encephalopathy, an indication with strong guideline support [33]. However, its use for non-infectious conditions poses a challenge for antibiotic stewardship programs that rely on systems like the AWaRe classification, which primarily focuses on infectious diseases [34]. This highlights a need for stewardship metrics that can appropriately evaluate non-standard uses of antibiotics. Our data also showed that levofloxacin, which comprised 7.8% of prescriptions, had a concerning susceptibility rate of less than 80% for *E. coli* in outpatients and approximately 10% for ESBL-producing strains. This low susceptibility rate, coupled with the absence of a significant increase in resistance during our observation period, may serve as a positive indicator of the effectiveness of our AST-based interventions. However, the high resistance levels for ESBL-producing strains still warrant caution, suggesting that levofloxacin should be reserved for specific cases where susceptibility is confirmed or when guided by strong clinical pathways, such as for febrile neutropenia, where patient compliance and established guidelines are favorable [35]. The observed resistance patterns, therefore, underscore the importance of integrating susceptibility data directly into prescribing decisions [32]. Similarly, roxithromycin and minocycline, comprising 7.0% and 5.3% of prescriptions, respectively, are often prescribed for long-term oral use for acne vulgaris. Based on guidelines, these can be changed to doxycycline, which has a higher recommendation level [36]. The need for the safety monitoring of doxycycline due to specific gastrointestinal adverse events, and its use in treating acne-like rashes triggered by tyrosine kinase inhibitors indicated for patients with non-small cell lung cancer and others, highlights the value of modifying clinical pathways and leveraging specialized teams to optimize prescription practices [37].

The analysis of specific antibiotic shifts provides further evidence of the intervention’s effect. Following the recommendations in dermatology to switch from Watch antibiotics like minocycline and roxithromycin to the Access antibiotic doxycycline, we observed a corresponding increase in doxycycline use (Supplementary Material, Table S1). Similarly, recommendations to use Access antibiotics like amoxicillin and amoxicillin/clavulanic acid for sinusitis and otitis media contributed to the overall shift (Supplementary Material, Table S1). The observation that the increase in DDDs for Access antibiotics (+ 376.5) was larger than the decrease for Watch antibiotics (-74.1) does not necessarily indicate an overall increase in antibiotic consumption. This could be explained by a shift from Watch antibiotics with lower standard DDDs that are often used for long durations (e.g., macrolides for their immunomodulatory effects [27]) to Access antibiotics used for shorter, acute infectious diseases. This highlights the complexity of interpreting consumption metrics and suggests that both DDDs and DOTs should be considered in tandem.

This study has several limitations. Its single-center, retrospective design and short duration limit the generalizability of findings and our ability to detect long-term resistance trends. Therefore, multi-center studies are warranted to validate our results across different settings. Additionally, the study did not capture all potential confounding factors, such as disease severity or comorbidities, which may have influenced prescribing decisions. Finally, the AWaRe classification system has limitations for evaluating antibiotics used in non-infectious conditions, which may have affected our results. Achieving sustained improvement in antibiotic stewardship necessitates a more comprehensive, multi-faceted approach. Proposed future strategies include the integration of disease-specific indicators for more precise evaluation, the implementation of real-time electronic health record alerts for prescribers, the strengthening of rapid diagnostic procedures to identify pathogens and resistance profiles faster, and the creation of a robust feedback loop connecting surveillance data, like local antibiograms, directly to physicians.

## Conclusions

Based on our AST interventions, we observed that a tailored educational approach could effectively modify outpatient prescribing habits, shifting usage from Watch to Access antibiotics. However, this success was limited to drug choice, with other factors including therapy duration unchanged. While resistance for specific agents like levofloxacin and sulfamethoxazole/trimethoprim showed a positive trend, a holistic assessment of resistance is essential. Future stewardship efforts require a more comprehensive strategy to address these challenges.

## Supplementary Information

Below is the link to the electronic supplementary material.

Supplementary Material 1

## Data Availability

The datasets for this article are not publicly available due to privacy concerns but are available from the corresponding author on reasonable request.

## Abbreviations

AMR: Antimicrobial resistance
AWaRe: Access, Watch, Reserve
WHO: World Health Organization
ESBL: Extended-spectrum β-lactamase
CLSI: Clinical and Laboratory Standards Institute
ATC: Anatomical therapeutic chemical
DDDs: Defined daily doses
DOTs: Days of therapy
TDD: Total daily dose
AST: Antimicrobial stewardship team
