# Supplementary material for: Impact of a tailored, department-specific antimicrobial stewardship team intervention based on AWaRe guidelines: a single-center, cohort, interrupted-time series study
Source: J Pharm Health Care Sci. 2025 Dec 25;12:11. doi: 10.1186/s40780-025-00525-3 (PMC12849487; doi:10.1186/s40780-025-00525-3)
Supplement: Supplementary file 1 — Supplementary Material 1 [file 40780_2025_525_MOESM1_ESM.docx]

Table S1. Detailed DDDs of antibiotics in the AWaRe classification (April 2022 to June 2025)

| AWaRe  classification | Generic name | FY  2022 Q1 | FY  2022 Q2 | FY  2022 Q3 | FY  2022 Q4 | FY  2023 Q1 | FY  2023 Q2 | FY  2023 Q3 | FY  2023 Q4 | FY  2024 Q1 | FY  2024 Q2 | FY  2024 Q3 | FY  2024 Q4 | FY  2025 Q1 |
| --- | --- | --- | --- | --- | --- | --- | --- | --- | --- | --- | --- | --- | --- | --- |
| Access | Sulfamethoxazole/  trimethoprim | 5,830 | 5,860 | 5,964 | 5,699 | 6,107 | 6,281 | 6,411 | 6,161 | 6,323 | 6,337 | 6,472 | 6,194 | 5,714 |
| Access | Doxycycline | 2,137 | 2,229 | 2,151 | 2,399 | 2,336 | 1,768 | 1,992 | 2,285 | 2,233 | 2,616 | 2,697 | 3,863 | 3,538 |
| Access | Amoxicillin | 663 | 602 | 612 | 668 | 594 | 782 | 857 | 615 | 756 | 791 | 899 | 805 | 823 |
| Access | Amoxicillin/  clavulanic acid | 240 | 295 | 295 | 323 | 254 | 382 | 261 | 261 | 398 | 387 | 527 | 508 | 910 |
| Access | Cefalexin | 239 | 210 | 262 | 184 | 268 | 258 | 204 | 169 | 209 | 238 | 241 | 192 | 245 |
| Access | Sultamicillin | 186 | 185 | 146 | 178 | 207 | 155 | 148 | 192 | 136 | 67 | 79 | 39 | 86 |
| Access | Clindamycin | 130 | 80 | 104 | 34 | 46 | 79 | 192 | 126 | 105 | 88 | 53 | 53 | 49 |
| Access | Metronidazole | 29 | 40 | 18 | 16 | 65 | 55 | 93 | 47 | 29 | 41 | 41 | 40 | 59 |
| Watch | Clarithromycin | 7,561 | 7,422 | 6,782 | 7,216 | 6,823 | 6,717 | 7,789 | 6,164 | 6,948 | 7,147 | 7,356 | 7,227 | 5,885 |
| Watch | Rifaximin | 2,540 | 2,723 | 2,230 | 2,947 | 3,428 | 3,654 | 4,008 | 3,494 | 3,800 | 3,952 | 4,256 | 3,642 | 3,488 |
| Watch | Roxithromycin | 2,208 | 1,918 | 1,850 | 1,485 | 1,799 | 2,270 | 2,176 | 2,598 | 2,324 | 2,421 | 2,562 | 2,384 | 2,185 |
| Watch | Levofloxacin | 2,036 | 2,628 | 2,222 | 2,022 | 2,297 | 2,588 | 2,399 | 2,154 | 2,132 | 1,736 | 1,949 | 1,359 | 1,735 |
| Watch | Erythromycin | 1,479 | 1,501 | 1,527 | 1,546 | 1,579 | 1,605 | 1,386 | 1,280 | 1,318 | 1,404 | 1,384 | 1,380 | 1,343 |
| Watch | Minocycline | 1,281 | 1,434 | 1,841 | 1,526 | 1,400 | 1,410 | 1,582 | 1,825 | 1,248 | 1,012 | 843 | 653 | 801 |
| Watch | Cefaclor | 679 | 706 | 618 | 471 | 583 | 721 | 620 | 665 | 636 | 820 | 716 | 698 | 847 |
| Watch | Azithromycin | 140 | 185 | 157 | 196 | 156 | 344 | 187 | 310 | 289 | 328 | 533 | 472 | 488 |
| Watch | Sitafloxacin | 217 | 296 | 272 | 225 | 182 | 303 | 357 | 350 | 362 | 218 | 198 | 166 | 334 |
| Watch | Cefcapene | 299 | 377 | 222 | 241 | 197 | 145 | 169 | 152 | 146 | 188 | 225 | 128 | 205 |
| Watch | Lascufloxacin | 68 | 19 | 29 | 85 | 98 | 84 | 84 | 192 | 160 | 127 | 154 | 117 | 72 |
| Watch | Fosfomycin | 90 | 58 | 74 | 82 | 73 | 108 | 121 | 87 | 114 | 162 | 130 | 96 | 76 |
| Watch | Garenoxacin | 71 | 45 | 58 | 70 | 108 | 62 | 61 | 53 | 102 | 68 | 124 | 158 | 158 |
| Watch | Cefdinir | 77 | 99 | 78 | 55 | 70 | 71 | 87 | 72 | 51 | 56 | 62 | 81 | 82 |
| Watch | Cefpodoxime | 101 | 102 | 117 | 66 | 67 | 76 | 86 | 86 | 53 | 47 | 36 | 33 | 38 |
| Watch | Kanamycin | 77 | 81 | 86 | 37 | 83 | 102 | 50 | 58 | 79 | 70 | 63 | 46 | 28 |
| Watch | Cefditoren | 74 | 57 | 66 | 46 | 52 | 63 | 44 | 101 | 35 | 49 | 76 | 42 | 22 |
| Watch | Ciprofloxacin | 21 | 15 | 9 | 2 | 16 | 31 | 31 | 6 | 25 | 61 | 28 | 35 | 51 |
| Watch | Prulifloxacin | 8 | 23 | 0 | 0 | 3 | 12 | 28 | 23 | 20 | 28 | 38 | 6 | 5 |
| Watch | Tosufloxacin | 20 | 6 | 6 | 6 | 0 | 6 | 4 | 13 | 8 | 19 | 19 | 0 | 1 |
| Watch | Fidaxomicin | 0 | 0 | 0 | 0 | 0 | 0 | 10 | 0 | 0 | 0 | 0 | 0 | 0 |
| Watch | Tebipenem | 0 | 1 | 0 | 0 | 0 | 0 | 0 | 0 | 0 | 0 | 0 | 0 | 0 |
| Reserve | Faropenem | 30 | 18 | 51 | 36 | 34 | 53 | 24 | 53 | 102 | 81 | 53 | 85 | 24 |
| Reserve | Linezolid | 0 | 7 | 12 | 35 | 0 | 0 | 0 | 27 | 24 | 0 | 0 | 0 | 3 |
| Reserve | Tedizolid | 0 | 0 | 0 | 0 | 0 | 0 | 0 | 0 | 0 | 0 | 0 | 0 | 0 |

Table S2. Detailed DOTs of antibiotics in the AWaRe classification (April 2022 to June 2025)

| AWaRe  classification | Generic name | FY  2022 Q1 | FY  2022 Q2 | FY  2022 Q3 | FY  2022 Q4 | FY  2023 Q1 | FY  2023 Q2 | FY  2023 Q3 | FY  2023 Q4 | FY  2024 Q1 | FY  2024 Q2 | FY  2024 Q3 | FY  2024 Q4 | FY  2025 Q1 |
| --- | --- | --- | --- | --- | --- | --- | --- | --- | --- | --- | --- | --- | --- | --- |
| Access | Sulfamethoxazole/  trimethoprim | 22,807 | 23,191 | 23,524 | 22,966 | 24,591 | 25,271 | 25,615 | 24,876 | 25,116 | 25,204 | 25,018 | 24,387 | 22,258 |
| Access | Doxycycline | 1,938 | 1,841 | 1,736 | 1,825 | 1,895 | 1,466 | 1,658 | 2,003 | 1,997 | 2,342 | 2,510 | 3,167 | 2,979 |
| Access | Amoxicillin | 1,136 | 1,187 | 1,246 | 1,290 | 1,218 | 1,531 | 1,844 | 1,397 | 1,697 | 1,779 | 1,954 | 1,708 | 1,763 |
| Access | Amoxicillin/  clavulanic acid | 488 | 564 | 632 | 639 | 488 | 742 | 495 | 497 | 756 | 743 | 1,041 | 1,002 | 921 |
| Access | Cefalexin | 677 | 586 | 626 | 433 | 585 | 579 | 446 | 384 | 502 | 498 | 553 | 470 | 565 |
| Access | Sultamicillin | 358 | 336 | 256 | 300 | 316 | 237 | 204 | 324 | 245 | 103 | 107 | 52 | 115 |
| Access | Clindamycin | 259 | 215 | 211 | 91 | 119 | 191 | 377 | 331 | 259 | 221 | 122 | 122 | 129 |
| Access | Metronidazole | 83 | 99 | 48 | 45 | 124 | 140 | 162 | 110 | 76 | 101 | 106 | 115 | 158 |
| Watch | Clarithromycin | 8,387 | 7,963 | 7,562 | 8,581 | 8,592 | 8,647 | 9,828 | 7,777 | 8,569 | 8,212 | 8,753 | 8,573 | 7,293 |
| Watch | Erythromycin | 4,519 | 4,431 | 4,334 | 4,255 | 4,425 | 4,244 | 3,674 | 3,774 | 3,478 | 3,819 | 3,745 | 3,715 | 3,440 |
| Watch | Roxithromycin | 2,253 | 2,010 | 1,910 | 1,532 | 1,863 | 2,376 | 2,256 | 2,657 | 2,410 | 2,585 | 2,683 | 2,452 | 2,318 |
| Watch | Levofloxacin | 2,146 | 2,716 | 2,306 | 2,126 | 2,393 | 2,659 | 2,468 | 2,206 | 2,220 | 1,820 | 2,096 | 1,507 | 1,860 |
| Watch | Rifaximin | 1,375 | 1,500 | 1,250 | 1,595 | 2,008 | 2,261 | 2,302 | 2,173 | 2,292 | 2,536 | 2,572 | 2,591 | 2,290 |
| Watch | Minocycline | 1,664 | 1,816 | 1,773 | 1,935 | 1,810 | 1,890 | 2,025 | 2,277 | 1,638 | 1,211 | 1,131 | 858 | 1,093 |
| Watch | Cefaclor | 1,666 | 1,629 | 1,448 | 1,168 | 1,644 | 1,592 | 1,391 | 1,046 | 1,184 | 1,694 | 1,355 | 1,470 | 1,848 |
| Watch | Sitafloxacin | 219 | 296 | 272 | 228 | 182 | 303 | 357 | 350 | 368 | 218 | 198 | 160 | 340 |
| Watch | Azithromycin | 45 | 54 | 60 | 120 | 81 | 194 | 188 | 334 | 315 | 361 | 587 | 525 | 544 |
| Watch | Cefcapene | 449 | 569 | 333 | 363 | 298 | 223 | 260 | 233 | 231 | 284 | 340 | 199 | 316 |
| Watch | Fosfomycin | 178 | 120 | 147 | 151 | 139 | 166 | 245 | 143 | 230 | 276 | 250 | 177 | 150 |
| Watch | Cefpodoxime | 226 | 222 | 259 | 176 | 157 | 185 | 206 | 204 | 114 | 130 | 90 | 84 | 86 |
| Watch | Lascufloxacin | 68 | 19 | 29 | 85 | 98 | 84 | 84 | 192 | 160 | 127 | 154 | 117 | 72 |
| Watch | Cefdinir | 160 | 211 | 158 | 110 | 154 | 148 | 178 | 143 | 108 | 114 | 123 | 172 | 171 |
| Watch | Kanamycin | 154 | 161 | 172 | 74 | 165 | 203 | 99 | 116 | 158 | 140 | 126 | 91 | 56 |
| Watch | Garenoxacin | 71 | 45 | 60 | 70 | 108 | 62 | 69 | 53 | 102 | 70 | 131 | 158 | 159 |
| Watch | Cefditoren | 112 | 81 | 97 | 60 | 77 | 72 | 61 | 133 | 50 | 67 | 104 | 62 | 33 |
| Watch | Ciprofloxacin | 52 | 33 | 18 | 3 | 33 | 49 | 35 | 14 | 35 | 93 | 35 | 84 | 66 |
| Watch | Tosufloxacin | 30 | 7 | 7 | 7 | 0 | 11 | 5 | 33 | 10 | 35 | 24 | 0 | 5 |
| Watch | Prulifloxacin | 12 | 35 | 0 | 0 | 5 | 18 | 16 | 3 | 9 | 18 | 7 | 2 | 8 |
| Watch | Fidaxomicin | 0 | 0 | 0 | 0 | 0 | 0 | 10 | 0 | 0 | 0 | 0 | 0 | 0 |
| Watch | Tebipenem | 0 | 3 | 0 | 0 | 0 | 0 | 0 | 0 | 0 | 0 | 0 | 0 | 0 |
| Reserve | Faropenem | 38 | 22 | 73 | 50 | 43 | 66 | 31 | 66 | 133 | 106 | 73 | 106 | 31 |
| Reserve | Linezolid | 0 | 7 | 12 | 35 | 0 | 0 | 0 | 27 | 24 | 0 | 0 | 0 | 7 |
| Reserve | Tedizolid | 0 | 0 | 0 | 0 | 0 | 0 | 0 | 0 | 0 | 0 | 0 | 0 | 0 |

Table S3. Detailed number of outpatients receiving antibiotics in the AWaRe classification (April 2022 to June 2025)

| AWaRe  classification | Generic name | FY  2022 Q1 | FY  2022 Q2 | FY  2022 Q3 | FY  2022 Q4 | FY  2023 Q1 | FY  2023 Q2 | FY  2023 Q3 | FY  2023 Q4 | FY  2024 Q1 | FY  2024 Q2 | FY  2024 Q3 | FY  2024 Q4 | FY  2025 Q1 |
| --- | --- | --- | --- | --- | --- | --- | --- | --- | --- | --- | --- | --- | --- | --- |
| Access | Sulfamethoxazole/  trimethoprim | 1,310 | 1,312 | 1,387 | 1,403 | 1,489 | 1,518 | 1,509 | 1,479 | 1,517 | 1,456 | 1,498 | 1,498 | 1,347 |
| Access | Amoxicillin | 105 | 113 | 122 | 118 | 117 | 151 | 141 | 107 | 135 | 140 | 147 | 156 | 147 |
| Access | Amoxicillin/  clavulanic acid | 58 | 59 | 71 | 68 | 58 | 80 | 66 | 57 | 77 | 76 | 87 | 93 | 180 |
| Access | Cefalexin | 91 | 91 | 80 | 64 | 92 | 82 | 76 | 60 | 71 | 57 | 65 | 59 | 59 |
| Access | Doxycycline | 50 | 43 | 42 | 44 | 48 | 39 | 50 | 57 | 53 | 70 | 70 | 95 | 84 |
| Access | Sultamicillin | 19 | 17 | 18 | 17 | 30 | 30 | 25 | 21 | 18 | 11 | 11 | 7 | 7 |
| Access | Metronidazole | 11 | 14 | 6 | 6 | 10 | 15 | 12 | 12 | 9 | 12 | 14 | 10 | 17 |
| Access | Clindamycin | 8 | 4 | 8 | 1 | 3 | 5 | 11 | 8 | 6 | 8 | 6 | 5 | 5 |
| Watch | Levofloxacin | 275 | 292 | 250 | 265 | 302 | 300 | 268 | 255 | 257 | 247 | 253 | 198 | 244 |
| Watch | Clarithromycin | 174 | 159 | 155 | 183 | 190 | 189 | 213 | 177 | 188 | 180 | 219 | 201 | 180 |
| Watch | Cefaclor | 115 | 119 | 107 | 93 | 107 | 118 | 115 | 111 | 113 | 155 | 142 | 121 | 128 |
| Watch | Erythromycin | 81 | 80 | 79 | 81 | 77 | 67 | 64 | 70 | 59 | 70 | 66 | 64 | 60 |
| Watch | Minocycline | 58 | 74 | 76 | 67 | 64 | 65 | 76 | 95 | 63 | 58 | 46 | 41 | 50 |
| Watch | Cefcapene | 81 | 80 | 73 | 78 | 67 | 54 | 59 | 59 | 57 | 58 | 64 | 42 | 50 |
| Watch | Roxithromycin | 51 | 46 | 41 | 35 | 45 | 61 | 54 | 63 | 56 | 65 | 67 | 67 | 66 |
| Watch | Rifaximin | 27 | 37 | 33 | 37 | 49 | 47 | 44 | 44 | 46 | 47 | 49 | 43 | 47 |
| Watch | Cefdinir | 30 | 35 | 26 | 21 | 23 | 24 | 23 | 25 | 19 | 19 | 20 | 27 | 25 |
| Watch | Cefpodoxime | 36 | 32 | 38 | 27 | 22 | 19 | 25 | 21 | 18 | 15 | 13 | 12 | 11 |
| Watch | Azithromycin | 10 | 17 | 18 | 18 | 16 | 18 | 21 | 27 | 21 | 22 | 29 | 24 | 24 |
| Watch | Cefditoren | 25 | 18 | 26 | 16 | 22 | 16 | 15 | 24 | 12 | 15 | 22 | 19 | 10 |
| Watch | Fosfomycin | 13 | 8 | 10 | 9 | 8 | 13 | 17 | 16 | 21 | 25 | 24 | 13 | 14 |
| Watch | Sitafloxacin | 11 | 11 | 13 | 11 | 11 | 11 | 11 | 16 | 21 | 14 | 13 | 11 | 18 |
| Watch | Lascufloxacin | 10 | 3 | 4 | 12 | 14 | 11 | 12 | 19 | 18 | 16 | 22 | 16 | 11 |
| Watch | Garenoxacin | 11 | 6 | 9 | 11 | 11 | 9 | 10 | 8 | 12 | 10 | 14 | 20 | 10 |
| Watch | Kanamycin | 5 | 5 | 5 | 2 | 4 | 5 | 4 | 4 | 5 | 5 | 4 | 2 | 1 |
| Watch | Ciprofloxacin | 3 | 4 | 2 | 1 | 3 | 4 | 2 | 1 | 3 | 7 | 3 | 6 | 6 |
| Watch | Prulifloxacin | 2 | 2 | 0 | 0 | 1 | 2 | 5 | 3 | 4 | 5 | 3 | 2 | 1 |
| Watch | Tosufloxacin | 3 | 1 | 1 | 1 | 0 | 2 | 1 | 3 | 2 | 5 | 3 | 0 | 1 |
| Watch | Tebipenem | 0 | 1 | 0 | 0 | 0 | 0 | 0 | 0 | 0 | 0 | 0 | 0 | 0 |
| Watch | Fidaxomicin | 0 | 0 | 0 | 0 | 0 | 0 | 1 | 0 | 0 | 0 | 0 | 0 | 0 |
| Reserve | Faropenem | 7 | 4 | 10 | 9 | 8 | 11 | 8 | 10 | 14 | 8 | 11 | 9 | 4 |
| Reserve | Linezolid | 0 | 1 | 1 | 1 | 0 | 0 | 0 | 1 | 2 | 0 | 0 | 0 | 1 |
| Reserve | Tedizolid | 0 | 0 | 0 | 0 | 0 | 0 | 0 | 0 | 0 | 0 | 0 | 0 | 0 |
